# Qiliqiangxin Capsule Improves Cardiac Function and Attenuates Cardiac Remodeling by Upregulating miR-133a after Myocardial Infarction in Rats

**DOI:** 10.1155/2019/7528214

**Published:** 2019-03-14

**Authors:** Huiyang Chen, Lixia Lou, Dongmei Zhang, Yizhou Zhao, Jing Zhao, Chunhong Li, Ya Huang, Keke Liu, Mingjing Zhao, Aiming Wu

**Affiliations:** Dongzhimen Hospital Affiliated to Beijing University of Chinese Medicine, Key Laboratory of Chinese Internal Medicine of Ministry of Education and Beijing, Beijing 100700, China

## Abstract

Qiliqiangxin capsule (QLC), a natural herb recipe with therapeutic effect from China, has been widely used in clinical practice for attenuating cardiac remodeling induced by myocardial infarction (MI). However, the pharmacological mechanism of QLC on cardiac remodeling after MI is not entirely clear. The present study aims to investigate the effectiveness and mechanisms of QLC on cardiac remodeling induced by MI in rats. The animal model was established by permanently ligating the left anterior descending coronary artery in rats. Subsequently, rats with successful ligation were randomly divided into model group, captopril group, and QLC group. And the control group was operated upon in parallel except ligation, namely, the sham group. All rats were treated through the intragastric administration once a day for 4 weeks. Cardiac hemodynamics was measured after treatment. Then, the left ventricular mass index (LVMI) was examined. The pathological changes were observed by HE staining. The collagen volume fraction (CVF) was detected by Masson trichrome staining. The apoptosis index was obtained by TUNEL fluorescent staining. The miR-133a and mRNA of TGF-*β*1, CTGF, Caspase9, and Caspase3 were examined by real-time PCR. The protein expressions of TGF-*β*1, CTGF, Caspase9, Caspase3, and cleaved-Caspase3 were tested by Western blot. Compared with the model group, QLC partially improved cardiac hemodynamics and decreased LVMI. miR-133a was significantly increased in QLC group. In addition, QLC declined CVF by downregulating TGF-*β*1 rather than CTGF. Meanwhile, QLC decreased the apoptosis index by attenuating Caspase9, Caspase3, and cleaved-Caspase3. This study suggested that QLC could improve cardiac function and partially attenuate cardiac remodeling by attenuating fibrosis and decreasing apoptosis, which might be partially related to miR-133a, TGF-*β*1, Caspase9, and Caspase3.

## 1. Introduction

Coronary heart disease is considered as one cardiovascular disease (CVD) seriously affecting human health worldwide, which may develop into myocardial infarction (MI) or even chronic heart failure. Recently, one study from Beijing, the capital of China, showed an increase in MI patients, and the authors called for more efforts to prevent it [1]. As we all know, cardiac remodeling after MI involves complex pathological changes, which cause cardiac dysfunction [2]. With the continuous exploration of pathological mechanism, people gradually realized the important roles of miRNAs in cardiac remodeling after MI [3, 4]. miRNAs, a class of small noncoding RNAs widely existing in vivo, regulate protein-coding genes and may be a suppressor of heart diseases [5]. Among them, miR-133a seemed to a central role in the diagnosis and the treatment of cardiac remodeling [6, 7]. Traditional Chinese medicine (TCM) has long been widely applied to treat CVD [8]. Whether its therapeutic mechanisms are related to miR-133a is a meaningful issue worthy of attention and discussion. At the same time, according to the TargetScan database (online database for predicting miRNA target genes), TGF-*β*1, CTGF, Caspase9, and Caspase3 might be downstream genes of miR-133a, and their changes also deserved our attention.

Ethnopharmacology is of extreme importance for the conservation and enhancement of TCM. Exploring and dedicating the pharmacological effects of TCM are of great significance to develop novel drugs for treating MI. The “Qi and Blood Theory” is the characteristic theory of TCM in dealing with MI. Founded on the theory of TCM and clinical practice, under normal conditions, the “Qi” and “Blood” are filled and circulate well in the human body to maintain life activities. However, “Qi” and “Blood” are abnormal (namely “Qi Deficiency and Blood Stasis”) after MI in the whole process of the disease. Benefiting Qi and activating blood circulation are the basic principles for treating MI in TCM. Qiliqiangxin capsule (QLC) is a natural herb recipe from China (Yiling Pharmaceutical Corporation), which is specially developed for benefiting Qi and activating blood circulation. And QLC has been approved by China Food and Drug Administration and widely used in clinic [9, 10]. However, the cardioprotective effects and mechanisms of this herb recipe have not been fully elucidated yet. The present study focuses on verifying the pharmacological effectiveness of QLC on cardiac remodeling after MI in rats and exploring the potential mechanisms of QLC on miR-133a, TGF-*β*1, CTGF, Caspase9, and Caspase3.

## 2. Materials and Methods

### 2.1. Ethics Statement

All experimental protocols and application of animals in this experiment were approved by the Standing Committee on Animals at the Dongzhimen Hospital Affiliated to Beijing University of Chinese Medicine. The SPF level of male Sprague-Dawley rats (weighted 200 ± 20 g) in this study was acquired from Beijing Vital River Laboratory Animal Technology Co. Ltd. (License number SCXK (Beijing) 2012-0001). During the experiment, all rats were raised in the animal barrier system of the Key Laboratory of Chinese Internal Medicine of the Ministry of Education and Beijing and with free access to deionized water and common feed.

### 2.2. Coronary Artery Ligation Surgery

The rats with MI were established by permanent left coronary artery ligation as previously described [11]. 1% pentobarbital sodium (5 ml/Kg) was used to anaesthetize rats by intraperitoneal injection. Then, in the supine position, tracheal intubation was performed in rats, and the rats were connected with the animal ventilator (ALC-V8S, Shanghai, China). Meanwhile, the twelve-lead electrocardiogram (ECG) was performed preoperatively (FX-7202, Beijing, China). Then, 2cm incision between the 3rd and 4th ribs on the left was made, and the subcutaneous tissue and muscle were separated carefully and bluntly. The left anterior descending coronary artery was ligated directly by 5/0 surgical line, while the sham operation group was assigned to the control group by only threading the surgical line without ligation. After the operations above, the thorax was tightly closed, and the ventilator was disconnected at the end of inhalation. All rats were carefully cared for until they regained sufficient consciousness to spontaneous breathing. Compared with preoperative ECG, corresponding leads showed ST-segment elevated immediately after ligation and the pathological Q-waves appeared on the second day after surgery, which indicated successful coronary artery ligation surgery. Finally, penicillin (F6062105, Hebei, China) was injected intraperitoneally for 3 days to prevent infection.

### 2.3. Allocation and Drug Administration

Rats with successful coronary artery ligation surgery were then randomly divided into model group, captopril group, and QLC group. Meanwhile, rats without ligation were set as the control group, namely, the sham group. Each group had 10 rats.

QLC (0.3g per capsule) consists of many natural herbs such as* Radix Astragali, Ginseng, Radix Aconiti Lateralis Preparata, Salviae Miltiorrhizae Radix, Semen Lepidii, Alismatis Rhizoma, Rhizoma Polygonati Odorati, Ramulus Cinnamomi, Carthami Flos, Cortex Periplocae, and Pericarpium Citri Tangerine*, which is the product of Shijiazhuang Yiling Pharmaceutical Co., Ltd (Shijiazhuang, China). The captopril tablet (12.5mg per table) is the product of Sino-American Shanghai Squibb Pharmaceuticals Ltd. (Shanghai, China). The drugs were thoroughly mixed with deionized water. Subsequently, according to the group, all rats were treated via intragastric administration (2.25 mg/Kg/d in the captopril group, 0.32 g/Kg/d in the QLC group, equal volume of deionized water in the model group and the sham group). The treatment lasted for 4 weeks from the second day after surgery (10 ml/Kg/d by gavage).

### 2.4. Measurements of Cardiac Hemodynamic

The intubation of internal left ventricular was carried out to detect cardiac hemodynamics in rats (BL-420S, Chengdu, China) [12]. 1% pentobarbital sodium (5 ml/Kg) was performed to anaesthetize rats by intraperitoneal injection after treatment. Then, after skin preparation and disinfection of the neck, the rats were positioned in supine position. The anterior cervical muscles were separated bluntly to expose the right carotid artery. A heparin-soaked PE catheter (0.05mm inner diameter) was inserted into the right carotid artery and carefully pushed to the left ventricle. The other end of the catheter was connected to the biological function acquisition system (BL-420S, Chengdu, China). After the signal stabilizes, recorded the following 4 indicators: left ventricular systolic pressure (LVSP), left ventricular end diastolic pressure (LVEDP), the maximal rate of increase or decrease of left ventricular pressure (±dp/dt max).

### 2.5. Left Ventricular Mass Index (LVMI)

After removing the rat hearts, we rinsed it completely in precooled 0.9% physiological saline. Then, we trimmed unnecessary parts off the heart, like the right atrium, right ventricle, left atrial appendage, and excess blood vessels. We recorded the Left Ventricle Mass (g, LVM) and Body Mass (g, BM). The LVMI equals the ratio of LVM to BM, LVMI= LVM/BM.

### 2.6. Pathological Staining Experiments

#### 2.6.1. H&E Staining

Histological samples were fixed in 4% paraformaldehyde for 24 hours, embedded in paraffin, and then made 4*μ*m section by drum-type slicer (RM2135, LEICA, Germany). Subsequently, they were deparaffinized through xylene and rehydrated via a series of ethanol washes (100%, 95%, 90%, 80%, and 70% ethanol). The specific steps of H&E staining were as follows: H&E Staining Kit (D006, Nanjing, China), hematoxylin staining for 5min, deionized water washing for 10s, eosin staining for 2min, hyperchromic liquid washing for 10s×2, dehydrating by 100% ethanol for 30s and washing by xylene for 15min. Finally, observed the section with the microscope (Axio Scope.A1, ZEISS, Germany).

#### 2.6.2. Masson Trichrome Staining

Masson Trichrome Staining Kit (D026, Nanjing, China), 4-*μ*m-thick paraffin sections, hematoxylin staining for 1min, deionized water washing for 30s, eosin staining for 40s, deionized water washing for 30s, aniline blue staining for 2.5min, deionized water washing for 10s, dehydrating by 100% ethanol for 30s and washing by xylene for 15min, and sealing with resinous mounting medium (ZLI-9516, Beijing, China) were used. Finally, we observed sections with microscope (Axio Scope.A1, ZEISS, Germany). The collagen content was analyzed by Image-Pro Plus 6.0 (American), and the collagen volume fraction (CVF) equals the ratio of collagen area to the sum of myocardial area and collagen area, and the mean value represented the CVF of the section.

#### 2.6.3. TUNEL Assay

Prepared and pretreated 4-*μ*m-thick paraffin sections of myocardial tissues in strict accordance with the DeadEnd™ Fluorometric TUNEL System (G3250, Promega, American). We covered the tissue with 100*μ*l of Equilibration Buffer at room temperature (RT) for 10min. Meanwhile, 51*μ*l rTdT incubation buffers (consist of 45*μ*l Equilibration Buffer, 5*μ*l Nucleotide Mix, 1*μ*l rTdT Enzyme) were strictly prepared and temporarily stored on ice for each slice under dark conditions. Then, they were blotted around the equilibrated areas with nonfiber paper to remove the Equilibration Buffer adding 51*μ*l of rTdT incubation buffer to the tissue. The operation of the negative control was the same as above but without rTdT Enzyme. After covering the tissue with cover glass, we incubated slides inside the humidified chamber under dark conditions for 60min at 37°C to allow the tailing reaction to occur. Next, we removed the cover glass and terminated the reactions by immersing the slides in 2X SSC for 15 min at RT. After that, we washed the slides in fresh PBS solution for 5min×3 to remove unincorporated fluorescein-12-dUTP. Finally, we added one drop of antifade solution with DAPI (S2110, Solarbio, American) on the myocardial tissue, covered the slides by cover glass, and sealed the edges with clear nail polish. Immediately we analyzed samples under a fluorescence microscope (Axio Scope.A1, ZEISS, Germany) and performed a standard fluorescein filter set to view the green fluorescence of fluorescein at 520nm, which indicated the apoptotic nucleus, and blue DAPI at 460nm, which was considered as all nucleus. The number of apoptotic nucleus and normal nucleus was counted by Image-Pro Plus 6.0 (American). We analyzed the apoptosis index of each group.

### 2.7. Real-Time PCR Analysis

The total RNA in the sample was extracted in strict accordance with the miRNA extraction kit (217004, QIAGEN Company, American) and Trizol regent (15596026, Thermo Fisher Scientific, American). We confirmed the ratio of OD 260/OD 280 in the range of 1.8-2.0. The primers sequences of miR-133a were obtained from Applied Biosystems (4427975, American). The primers sequences of TGF-*β*1, CTGF, Caspase9, and Caspase3 were synthesized by SinoGenoMax Co., Ltd. (Beijing, China). They were prepared for real-time PCR reaction system and amplified under the following conditions: miR-133a: predenatured at 94°C for 10min, denatured at 94°C for 15s, annealed at 60°C for 60s, extended at 72°C for 10s, and cycled above reaction for 45 times. The U6 was set as an internal control. Reaction conditions for TGF-*β*1, CTGF, Caspase9, and Caspase3 were as follows: predenatured at 95°C for 10 min, denatured at 95°C for 30s, annealed at 55°C for 30s, extended at 72°C for 20s, and circulated for 40 times. The *β*-actin was considered as internal control. The primer sequences were as follows:

miR-133a (F:5'-ATGGTTCGTGCGTTTGGTCCCCTTCAACC-3', R:5'-GCAGGGTCCGAGGTATTC-3'), U6 (F:5'-GCTTCGGCAGCACATATACTAAAAT-3', R:5'-CGCTTCACGAATTTGCGTGTCAT-3'). TGF-*β*1 (F: 5'-GCAACAACGCAATCTATGA-3', R: 5'-CAAGGTAACGCCAGGAAT-3'), CTGF (F: 5'-CTATGATGCGAGCCAACT-3', R:5'-CGGTAGGTCTTCACACTG-3'), Caspase9 (F: 5'-GCCACTGCCTCATCATCAACAA-3, R: 5'-TCGTTCTTCACCTCCACCATGA-3'), Caspase3 (F: 5'-GAATCCACGAGCAGAGTC-3', R: 5'-TCAACAAGCCAACCAAGT-3'), *β*-actin (F:5'-TACCCCATTGAACACGGCAT-3', R: 5'-AGGCATACAGGGACAACACA-3').

### 2.8. Western Blot

The myocardial tissue was lysed to obtain the supernatant. The protein concentration was detected by BCA method. Samples (30*μ*g/lane) were electrophoresed in 10% or 12% SDS-PAGE and transferred onto 0.2*μ*m NC membrane (A10161124, GE healthcare Life Science, American). The membranes were incubated with the primary antibodies overnight at 4°C and incubated with secondary antibody for 1 hour at RT. Strips were detected by Super ECL Plus and then analyzed with Image J.

### 2.9. Statistical Analysis

All values were analyzed with SPSS22.0 and presented as means ± standard deviation (SD). One-way ANOVA of LSD test was used for comparisons between multiple groups. Comparisons of nonnormal distribution data were followed by the Kruskal-Wallis method.* P* value < 0.05 was considered statistically significant.

## 3. Results

### 3.1. Change of QLC on Hemodynamic Assessments

**Figure 1:**
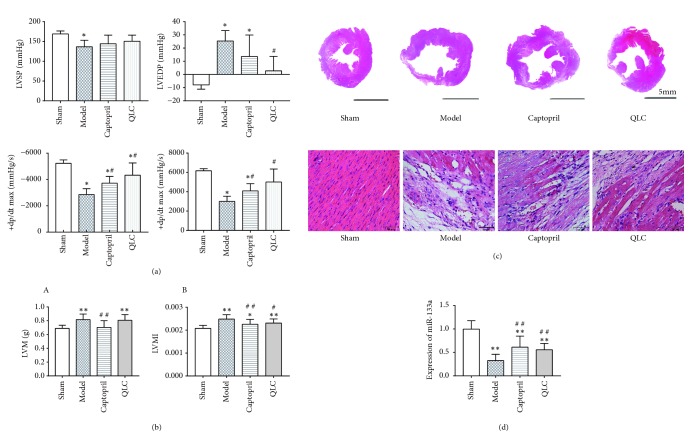
(a) Hemodynamic assessments (LVSP, LVEDP, and ±dp/dt max) of each group at 4 weeks after surgery. (b) H&E staining of each group at 4 weeks after surgery. (c) LVM and LVMI of each group at 4 weeks after surgery. (d) The expression level of miR-133a in the left ventricular myocardial tissue of each group at 4 weeks after surgery. The data are expressed as Mean ± SD. ^*∗*^*P*<0.05, ^*∗∗*^*P* <0.01 versus the sham group, ^#^*P*<0.05, ^# #^*P*<0.01 versus the model.

In vivo, cardiac hemodynamics could directly indicate the changes of left ventricular pressure in the whole cardiac cycle, which was always used to diagnose the systolic and diastolic functions of left ventricle. As shown in (Figure 1(a)), compared with the sham group, the LVSP significantly decreased in the model group and the captopril group. Compared with the model group, the LVSP had no change in the captopril and QLC group. Compared with the sham group, the LVEDP significantly increased while the ±dp/dt max was significantly decreased in model group. Compared with the model group, the LVEDP was significantly attenuated after treatment of QLC and captopril, and the ±dp/dt max was significantly improved in the captopril group and the QLC group.

### 3.2. Change of LVM and LVMI

LVM and LVMI were always used as indicators to roughly reflect the left ventricular remodeling. As shown in (Figure 1(b)), compared with the sham group, the LVM was significantly increased in the model group. When compared with the model group, it was partially decreased in the captopril group rather than the QLC group. When BM was considered, the results showed that the LVMI was significantly increased in the model group when compared with the sham group. Compared with the model group, the LVMI was partially decreased in the captopril group and the QLC group.

### 3.3. Change of QLC on Histopathology

H&E staining could partially reflect the changes of pathological structure of myocardial tissue at 4 weeks after surgery. As shown in (Figure 1(c)), when compared with the sham group, pathological tissue in the model group showed the left ventricular wall existed in a widely thinned and infarcted zone; meanwhile, the cavity of left ventricular increased obviously. These pathological changes were relatively improved in the captopril and QLC group. Similarly, under high magnification (40×), the myocardial cells in the model group were partially lost and disordered when compared with the sham group. However, after treatments of captopril and QLC, the shape of myocardial fibers in the infarct margin area was basically regular and the number of necrotic myocardial cells was decreased.

### 3.4. Change of miR-133a

In this part, the expression level of miR-133a was detected by real-time PCR reflected. As shown in (Figure 1(d)), the miR-133a was significantly decreased in the model group when compared with the sham group. However, compared with the model group, the expression levels of miR-133a were all increased in the captopril group and the QLC group.

### 3.5. Change of Myocardial Fibrosis

#### 3.5.1. Change of CVF

**Figure 2:**
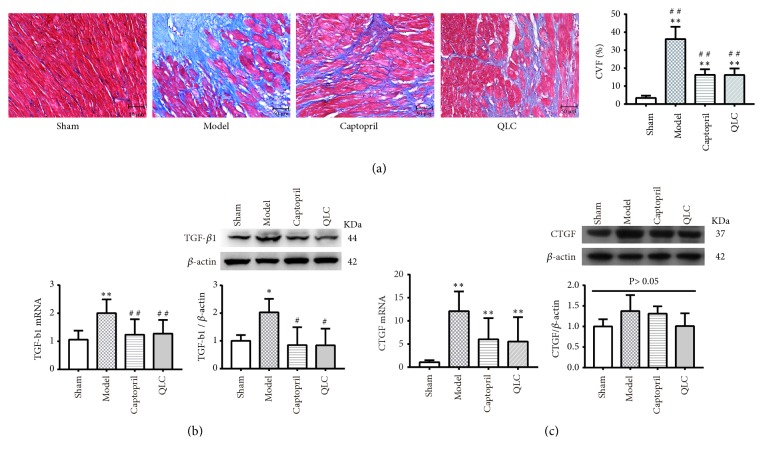
(a) Masson trichrome staining and the CVF of each group at 4 weeks after surgery. Blue represented collagen and red indicated normal myocardial tissue. (b) The mRNA and protein expression level of TGF-*β*1. (c) The mRNA and protein expression level of CTGF. The data are expressed as Mean ± SD. ^*∗*^*P*<0.05, ^*∗∗*^*P* <0.01 versus the sham group, ^#^*P*<0.05, ^# #^*P*<0.01 versus the model.

Based on the Masson trichrome staining, CVF was a recommended index for evaluating fibrosis of the left ventricular. As shown in (Figure 2(a)), normal myocardial tissue in the sham group had only a small amount of collagen. However, a large number of blue collagen occupied the infarcted and interstitial area of myocardial cells in the model group. After treatment of captopril and QLC, collagen was partially decreased. Compared with the sham group, the CVF significantly increased in the model group. Compared with the model group, the CVF was significantly attenuated in the captopril and QLC group.

#### 3.5.2. Changes of TGF-*β*1 and CTGF

TGF-*β*1 and CTGF were involved in the process of fibrosis, and their expressions were detected by real-time PCR and Western blot. As shown in (Figure 2(b)), compared with the sham group, the expression level of TGF-*β*1 mRNA was significantly increased in the model group. When compared with the model group, it was significantly decreased in the captopril and QLC group. Similarly, compared with the sham group, the protein expression level of TGF-*β*1 was increased in model group. When compared with the model group, it was attenuated in the captopril group and the QLC group. Compared with the sham group, the protein expression level of CTGF had no change although CTGF mRNA was increased in the model group. Meanwhile, whether at the gene or protein level, captopril and QLC showed no effect on CTGF when compared with the model group (Figure 2(c)).

### 3.6. Change of the Apoptotic Cardiomyocytes

#### 3.6.1. Change of the Apoptosis Index

**Figure 3:**
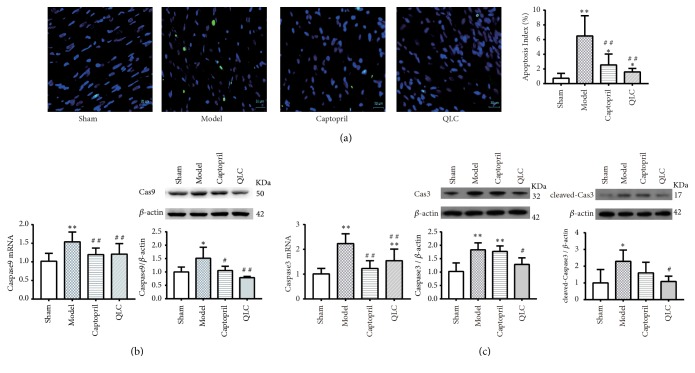
(a) TUNEL fluorescent staining and statistical analysis of apoptosis index in each group at 4 weeks after surgery. Green fluorescence indicated apoptotic nucleus and blue fluorescence indicated all nucleus. (b) The mRNA and protein expression level of Caspase9. (c) The mRNA and protein expression level of Caspase3 and the expression level of cleaved-Caspase3. The data are expressed as Mean ± SD. ^*∗*^*P*<0.05, ^*∗∗*^*P* <0.01 versus the sham group, ^#^*P*<0.05, ^# #^*P*<0.01 versus the model.

Since cardiomyocytes apoptosis was considered as important participants after MI. The Apoptosis Index, detected by the classical TUNEL assay, was an index for evaluating apoptosis. As shown in (Figure 3(a)), the green fluorescence indicated the broken DNA strand and blue DAPI considered as all nucleus. The model group elicited a significantly increased apoptotic nucleus in comparison with the sham group, which was reduced by captopril and QLC treatment. Precisely, compared with the sham group, the apoptosis index in model group was significantly increased. When compared with the model group, it was partially but significantly attenuated in the captopril group and the QLC group.

#### 3.6.2. Changes of Caspase9, Caspase3, and Cleaved-Caspase3

Caspase9 and Caspase3 were both important participants in apoptosis signaling pathway, whose expressions were tested by real-time PCR and Western blot in this part. Compared with the sham group, expression levels of Caspase9 mRNA were significantly increased in the model group (Figure 3(b)), and captopril and QLC could significantly decrease the level of Caspase9 mRNA when compared with the model group. At the protein level, Caspase9 had significantly increased in the model group when compared with the sham group, while the situation was partially suppressed by treatment of captopril and QLC when compared with the model group. In addition, compared with the model group, the level of Caspase3 mRNA was upregulated in the model group. While it was downregulated by treatment of captopril and QLC when compared with the model group (Figure 3(c)). At protein level, compared with the sham group, Caspase3 and cleaved-Caspase3 significantly increased in the model group. When compared with the model group, QLC could partially attenuate the expression level of Caspase3 and cleaved-Caspase3 while captopril failed to play a regulatory role in them.

## 4. Discussion

Cardiac remodeling after MI is a complex pathophysiological process that seriously threatens the patient's life, which includes inflammation, fibrosis, apoptosis, and other pathological processes. These pathological factors contribute to the development of heart failure after MI, which indicate that it is positive to mitigate the adverse impact as soon as possible [13]. In China, TCM have been considered as an alternative and adjuvant approach to the prevention of CVD and injury induced by ischemia/reperfusion [8, 14, 15]. The present study focuses on QLC, composed of multiple natural herbs, which plays a protective role in cardiac remodeling [16]. However, its pharmacological mechanism is not entirely clear. This study revealed QLC could partially improve cardiac hemodynamics and early ventricular remodeling after MI, and these pharmacological effects might partially attribute to its ability to regulate miR-133a, TGF-*β*1, Caspase9, and Caspase3.

In this experiment, firstly, the cardiac hemodynamics was carried out to evaluate the effect of QLC on cardiac function in vivo. The results showed that QLC could partially improve cardiac function in rats at 4 weeks after surgery. The effect of QLC on improving cardiac hemodynamics might rely on several main constituents in the compound. Ma et al. [17] suggested that the astragalus membranaceus dramatically improved cardiac function, including the ±dp/dt max, LVSP and LVEDP. Moreover, Zheng et al. [18] claimed that Astragaloside IV can induce ischemia/reperfusion injury and improve cardiac function. In addition, the Ginsenoside Rg3, one of the principal constituents in Panax ginseng, could significantly increase the left ventricular fractional shortening and improve the left ventricular ejection fraction in rats [19]. The recent study demonstrated that tanshinone IIA, the main component of salvia miltiorrhiza, increased the ejection fraction and ±dp/dt max in rats with MI [20]. In fact, the cardiac remodeling process induces the abnormal hemodynamics and further stress upon the left ventricular wall, inciting a vicious cycle of stress and remodeling [21]. In the present study, H&E staining and LVMI generally showed the pathological changes of heart after MI, and the results suggested that QLC decreased infarct zone and the LVMI after MI. Therefore, QLC could improve cardiac function and partially attenuate pathological changes in rats after MI.

Cardiac remodeling after MI was always accompanied by the complex gene regulation. miRNAs are considered as a group of small noncoding RNAs, and there are about one-third of human genes regulated by more than 2000 miRNAs [22]. These special miRNAs can negatively regulate the translation of target genes or degrade target genes after being processed and matured. miR-133a, one of the cardiac-specific miRNAs, is associated with cardiac hypertrophy, heart failure [23, 24]. Abdullatif M [25] revealed that the expression of miR-133 in myocardial hypertrophy model was significantly downregulated and negatively related to the degree of myocardial hypertrophy. Song et al. [26] found that the expression of miR-133a in the heart of chronic heart failure from patients or rats was decreased; however, overexpression of miR-133a improved cardiac hemodynamics and cardiac remodeling in chronic heart failure rats. In the present study, the expression level of miR-133a in infarct margin area was further detected. We found that miR-133a was significantly decreased because of MI-surgery while QLC could increase it. Therefore, upregulation of miR-133a might be one of the mechanisms of QLC on improving cardiac remodeling. After searching the database and previous studies, we found that TGF-*β*1, CTGF, Caspase9, and Caspase3 were closely related to miR-133a. The following work was mainly to explore and discuss their changes after MI as well as the intervention effects of QLC.

The complicated remodeling induced by MI is considered a mainly pathophysiological process consisting of multiple links, including oxygen deficit, myocardial fibrosis, and apoptosis. The fibrosis is always a key contributor during the development of cardiac dysfunction and remodeling in diverse pathological situations. In this study, QLC significantly decreased the CVF, which indicated that QLC could partially alleviate fibrosis after MI. As we all know, TGF-*β*1 and CTGF were recognized as cytokines that promote fibrosis. TGF-*β*1 could regulate extracellular matrix metabolism and initiate fibrosis [27]. Liu et al. [28] found that overexpression of miR-133 suppressed TGF-*β*1-mediated fibrosis. TGF-*β*1 might be a potential target gene for miR-133a. At the same time, Duisters et al. [29] found that miR-133 might combine with the 3' untranslated region of CTGF and thus downregulate CTGF. In addition, CTGF was also considered as the downstream mediator of the TGF-*β*1 pathway in cardiac fibroblasts and cardiac myocytes [30]. In this study, the results of real-time PCR and Western blot showed that QLC markedly decreased TGF-*β*1 while had no effect on CTGF. Therefore, the mechanism of QLC in alleviating fibrosis might be related to inhibit TGF-*β*1.

On the other hand, cardiomyocytes apoptosis was also involved in the pathological process of the left ventricular remodeling after MI. Early researches suggested that apoptosis had always been considered a programmed death process under physiological or pathological conditions, which were dispensable for physiological homeostasis in the normal organ [31]. However, it could also lead to excessive loss of myocardial cells that were associated with serious cardiac dysfunction in multiple pathological conditions, like the reperfusion injury, MI, and heart failure [32]. Abnormal changes or damages of mitochondria after MI were involved in apoptosis of cardiomyocytes in the infarcted area [2]. Cytochrome C and other apoptosis-promoting active proteins located in the gap between mitochondrial membranes are released into the cytoplasm, which mediated recruitment of pro-Caspase9 and eventually leaded to the activation of Caspase3 [2, 33]. Activation of Caspase3 was considered a necessary condition to initiate apoptosis. An early experiment had proved that cleavage of Caspase3 and apoptosis all decreased in Caspase9-knockout mice [34]. He et al. [35] suggested that miR-133 might inhibit apoptosis by targeting Caspase9. Meanwhile, when subjected to myocardial ischemia reperfusion injury, overexpression of Caspase3 increased infarct size and depressed cardiac function in mice [36]. Pharmacological intervention of apoptosis could diminish infarct area and improve cardiac function [37]. Song et al. [38] confirmed Caspase9 as the target of miR-133 and found the tanshinone IIA could reduce apoptosis by upregulation of miR-133 and downregulation of Caspase-9. Ginsenoside Rg3 might attenuate apoptosis through inhibiting the activation of Caspase3 in ischemia/reperfusion rats [19]. Similarly, in the rat model of myocardial ischemia, the astragalus membranaceus could enhance the myocardial cell viability via arresting the influx of Ca^2+^ to prevent apoptosis [17]. The Descurainia sophia (L.) Webb ex Prantl might improve cardiac function via regulating the balance between Bax and Bcl-2, blocking the activation of Caspase3 in the chronic heart failure rats [39]. Obviously, QLC contained many antiapoptosis components. This pharmacological effect of QLC as shown above might have a strong relationship with these components. In this study, QLC could attenuate Caspase9 and suppress Caspase3 thus protecting myocardial cells from apoptosis.

In conclusion, QLC could improve cardiac function and partially attenuate cardiac remodeling in rats after the ligation of left coronary artery. The pharmacological action of QLC on improving cardiac remodeling might be partially related to the reduction of fibrosis and apoptosis. And the underlying mechanism of QLC included increasing miR-133a, attenuating TGF-*β*1, Caspase9, Caspase3, and cleaved-Caspase3.

## Data Availability

The data used to support the findings of this study are available from the corresponding author upon request.
